# Using a customer discovery process to enhance the potential dissemination and scalability of a family healthy weight program for rural communities and small towns

**DOI:** 10.1186/s12966-024-01605-7

**Published:** 2024-05-14

**Authors:** Gwenndolyn C. Porter, Jennie L. Hill, Kate A. Heelan, R. Todd Bartee, Caitlin A. Golden, Ali Malmkar, Bryce A. Abbey, Paul A. Estabrooks

**Affiliations:** 1 University of Nebraska Medical Center, Department of Health Promotion, Omaha, NE 68198-4365 USA; 2 University of Utah, Population Health Sciences, Salt Lake City, UT 84108 USA; 3 University of Nebraska at Kearney, Kinesiology and Sport Sciences Department, Kearney, NE 68849 USA; 4 University of Nebraska at Kearney, Department of Biology, Kearney, NE 68849 USA; 5 University of Utah, Department of Health and Kinesiology, Salt Lake City, UT 84112 USA

## Abstract

**Aim:**

Customer discovery, an entrepreneurial and iterative process to understand the context and needs of potential adoption agencies, may be an innovative strategy to improve broader dissemination of evidence-based interventions. This paper describes the customer discovery process for the Building Healthy Families (BHF) Online Training Resources and Program Package (BHF Resource Package) to support rural community adoption of an evidence-based, family healthy weight program.

**Methods:**

The customer discovery process was completed as part of a SPeeding Research-tested INTerventions (SPRINT) training supported by the U.S. Centers for Disease Control and Prevention. Customer discovery interviews (*n*=47) were conducted with people that could be potential resource users, economic buyers, and BHF adoption influencers to capture multiple contextual and needs-based factors related to adopting new evidence-based interventions. Qualitative analyses were completed in an iterative fashion as each interview was completed.

**Results:**

The BHF Resource Package was designed to be accessible to a variety of implementation organizations. However, due to different resources being available in different rural communities, customer discovery interviews suggested that focusing on rural health departments may be a consistent setting for intervention adoption. We found that local health departments prioritize childhood obesity but lacked the training and resources necessary to implement effective programming. Several intervention funding approaches were also identified including (1) program grants from local and national foundations, (2) healthcare community benefit initiatives, and (3) regional employer groups. Payment plans recommended in the customer discovery interviews included a mix of licensing and technical support fees for BHF delivery organizations, potential insurance reimbursement, and family fees based on ability to pay. Marketing a range of BHF non-weight related outcomes was also recommended during the customer discovery process to increase the likelihood of BHF scale-up and sustainability.

**Conclusions:**

Engaging in customer discovery provided practical directions for the potential adoption, implementation, and sustainability of the BHF Resource Package. However, the inconsistent finding that health departments are both the ideal implementation organization, but also see childhood obesity treatment as a clinical service, is concerning.

## Background

The field of dissemination and implementation science has made pronounced strides in understanding barriers and facilitators related to translating efficacious interventions into practice [3]. Unfortunately, long lag times between the demonstration of intervention efficacy and use in community, public health, or healthcare settings persist [16]. Studying implementation processes and gaining an understanding of barriers to widespread uptake is a necessary, yet underutilized, tool in the pathway of moving interventions with demonstrated effectiveness into a position for broad adoption [23]. Further, the degree to which evidence-based interventions are moved into micropolitan (cities with <50,000 residents) and rural communities is an under-studied and high need area of research due to the confluence of lower socioeconomic status and health disparities [12].

Approaches such as user-centered design and community-academic partnerships attempt to speed the uptake of evidence-based interventions and close the research-practice gap [4, 6]. Within these approaches, designing for dissemination, equity, and sustainability at the beginning of the intervention development process is hypothesized to significantly increase the likelihood that the intervention will be successfully adopted, implemented with fidelity, and sustained [3, 5, 17] by considering the facilitators and barriers relevant to the intended implementation setting, delivery agent, and audience intended to benefit from an evidence-based intervention. User-centered design and partnerships approaches often use backward design processes to increase the likelihood that a given intervention fits the system characteristics based on the goal of adoption and sustainability [24]. This process includes identifying key outcomes that may result from the intervention, considering user groups’ needs, the selection of required content, and, finally, the mapping of intervention content and processes to key outcomes [5].

One strategy to improve broader dissemination of evidence-based interventions, that have similar underlying principles to user-centered design and participatory approaches, is the use of a customer discovery process [25]. Customer discovery focuses on generating motivational data from a range of key people or organizations that can help to refine intervention design and dissemination strategies, while providing insight on the potential for scalability. Funding organizations have applied customer discovery processes to support scientists that have developed effective interventions to determine a product-market fit for their work [7, 19]. Scientists involved in this process report seeing value in understanding customer segments and identifying value propositions that can guide dissemination efforts [7, 19]. This approach has also been successful in clarifying the product-market fit and in generating intervention adaptations to better serve relevant audiences [25]. Investigative teams also improved their familiarity and comfort with the market aspects of intervention delivery, resulting in a shift of focus towards implementation, pursuing connections with small businesses, establishing companies for intervention delivery, or, in some cases, redesigning interventions [19].

The Centers for Disease Control and Prevention (CDC) and the American Academy of Pediatrics (AAP) introduced an opportunity for research teams funded through the Childhood Obesity Research Demonstration (CORD) 3.0 project to participate in a facilitated customer discovery process. This process was adapted from the National Cancer Institute’s SPeeding Research-tested INTerventions (SPRINT) program [18] and was co-facilitated by the CDC and the AAP. All CORD 3.0 award recipients received training to facilitate the scale-up and acceptability of evidence-based, family healthy weight programs (FHWPs) with a goal to begin investigating sustainable funding and/or reimbursement structures. Participating research teams were encouraged to consider multiple customer segments – from families that could benefit from a FHWP, to community or clinical organizations that could contribute to implementation, and to agencies that could provide funding support.

This article reports on the application of a customer discovery process intended to improve future adoption, implementation, and sustainability of the Building Healthy Families (BHF) Online Training Resources and Program Package (BHF Resource Package; [10], the only CORD 3.0 project explicitly created to improve the uptake and delivery of a FHWP in rural and micropolitan areas [10, 11]. The BHF Resource Package was developed collaboratively by (1) researchers with expertise in community-engagement, family healthy weight program development, and implementation science, (2) Building Healthy Families (BHF) program developers and implementers, and (3) a community advisory board with representation from community, public health, and healthcare organizations. During this process, intended users, various user types, product needs, and anticipated problems were discussed and used to inform the BHF Resource Package development [10]. It was hypothesized that participation in the SPRINT training would generate innovative ideas for scaling up use of the BHF Resource Package. In this paper, we report on the processes and outcomes of a customer discovery process intended to document the unique characteristics of the BHF Resource Package marketplace in micropolitan and rural community settings, strategies to enhance adoption by community organizations, potential adaptations needed to improve the context-intervention fit, and potential sustainable funding models.

## Methods

Beginning in April through June of 2021, BHF team members (*n*=7) participated in a dissemination and implementation accelerator program, SPRINT, to improve the uptake of evidence based FHWPs. Our process focused on the BHF Resource Package which includes, but goes beyond the evidence-based BHF FHWP program, and has components focused on the development of sustainable program recruitment strategies and channels, training on general and session specific content for program coordinators, and an integrated data portal that includes knowledge checks, fidelity assessments, and parent and child behavioral and weight outcomes (all of which are used to generate community and family reports to demonstrate progress) [10, 11]. BHF SPRINT team members attended 10 meetings over the course of seven weeks and completed training in customer discovery, business modeling, interview techniques, and developing a translation/commercialization plan for the BHF Resource Package including dedicated modules on program revenue, costs, and market economics [18]. Each meeting included hands-on instruction on transforming evidence-based FHWPs into market-ready products and services [7]. All sessions were held synchronously using video conferencing to facilitate participation of CORD 3.0 research teams. This model allowed for peer sharing and learning across the other participating SPRINT research teams focused on translating FHWPs into sustained practice. SPRINT facilitators with expertise in customer discovery and business model planning held team-specific office hours biweekly to further develop and refine business model hypotheses. SPRINT teams generated business model hypotheses (e.g., how the BHF Resource Package would likely be disseminated and sustained in rural communities) to be tested through customer discovery interviews, which inform the creation of an operational business model – the SPRINT program’s final product. Hypotheses covered various segments of a business model, including key partners, key activities, key resources, value propositions, customer relations, communication channels, customer segments, cost structure, and revenue streams [20]. Details of the customer discovery interviews are outlined below.

### The BHF resource package

The BHF Resource Package was designed using participatory methods to address the variability in available organizations and personnel that could deliver BHF, an evidence-based FHWP that focuses on supporting family changes in behavioral skills, dietary changes, and physical activity promotion in micropolitan and rural areas [10]. The BHF Resource Package development included the identification of primary (i.e., facilitators to be trained to deliver BHF), secondary (i.e., researchers/evaluators interested in tracking outcomes and implementation quality), tertiary (i.e., organizations/individuals who would support/sponsor BHF), and terminal (i.e., BHF family participants) end users. Across each of these users groups the Reach, Effectiveness, Adoption, Implementation, and Maintenance (RE-AIM) Framework [8] was used, in a backwards-design process, to describe relevant outcomes. The resultant BHF Resource Package included (1) program materials, lesson plans, PowerPoint slides, and implementation checklists, (2) overarching and session specific training modules for program coordinators and facilitators [10], (3) knowledge checks to ensure coordinator and facilitator competence, (4) fidelity assessments to track implementation quality, (5) a recruitment module to support family engagement, and (6) a data portal to track program effectiveness and family progress.

### Participants

**Fig. 1:**
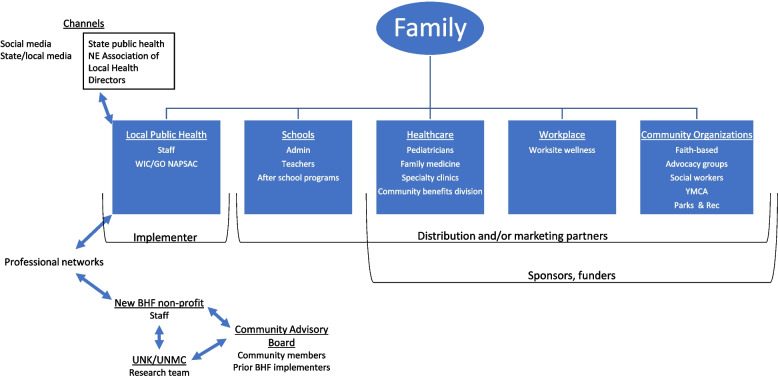
Ecosystem map for BHF - a family healthy weight program targeting rural and micropolitan communities

Community organizations, broadly defined as those with priorities related to childhood obesity and personnel with expertise or interest in family-based health promotion, were identified as the primary customer segment for the BHF Resource Package. This allowed for the consideration of community implementation team members from a broad range of community organizations (i.e., primary end users). Building from our primary customer segment, we created an eco-system map (Fig. 1) of rural FHWP. We used convenience sampling from existing professional networks to identify participants for our initial round of interviews. The eco-system map was generated to graphically represent the context of rural FHWP delivery and identify the relationships of individuals who would be involved in all aspects of the BHF Resource Package adoption, implementation, sustainment as well as those who would ultimately benefit from BHF program participation. The eco-system map identified potential customers and delivery channels (i.e., those who would support or influence BHF Resource Package implementation). The customer discovery process included interviews with individuals (*n*=47) representing different aspects of the eco-system map that could have an influence on, or interest in, the BHF Resource Package adoption and implementation in rural and micropolitan areas (Table 1). Interview findings are presented in aggregate (i.e., not separated by stakeholder group) as the formation of an operational business model requires generalizable fit across multiple stakeholder groups within a given eco-system.

**Table 1:** The role, customer type, and number of participants in the customer discovery interviews

**Customer Role**	**Customer Type**	**N**
Potential BHF Resource Package Users	Health Department	13
	Healthcare	10
	Worksite Wellness	4
	Schools	3
	Community Partner	2
Potential BHF Resource Package Economic Buyer/Payer	Public Health	3
	Healthcare	2
	Schools	2
	Worksite	2
Potential BHF Resource Package Influencers	Community Advocate	5
	Policy/Government	1
	Total	47

### Interviews

A broad approach with semi-structured, open-ended questions was taken with the interviews to explore and learn from potential BHF Resource Package adopters or influencers about their experiences related to addressing childhood obesity in their organization, identifying potential partners and competitors, and gaining insight about potential scale-up of BHF. The interviews were guided by an initial value proposition that was intended to incorporate key informant perceptions around things they would like to eliminate, things they would like to add, and considerations of the outcomes related to potential adoption of family healthy weight programs [20]. All participants were asked about how their organization prioritized or contributed to addressing childhood obesity, what other health promotion resources are available in the community, and community needs for addressing childhood obesity. Additional questions regarding program fit, such as, “If a childhood obesity program were to be offered by your organization, what would it look like?,” and, “What things would make it easier for your organization to offer this type of programming?” were asked during interviews. Discussions surrounding costs and revenue were central to all customer discovery interviews as the BHF SPRINT team sought to understand how best to create a sustainable pathway for the use of the BHF Resources Package and delivery of the FHWP in rural and micropolitan communities. Finally, our initial value proposition can be described as micropolitan and rural community organizations need and want to address childhood obesity, but currently do not have an easy way to do it. As such, we hypothesized that the BHF Resource Package would provide a valued, and relatively easy, turn-key approach to address childhood obesity locally.

The interview process followed an iterative approach – business model hypotheses were either confirmed – allowing testing of subsequent hypotheses – or refuted. When interview data suggested a hypothesis was incorrect or not feasible, then the business model hypotheses were revised to more closely align with the goal of creating a practical, robust, and compelling business model [19]. Of note, at the time of the interviews, the BHF Resource Package was being tested in seven micropolitan communities (and surrounding rural areas) in Nebraska with funding for program resources ($5,500/community) provided to participating communities through grant funding [9].

As the customer discovery interviews were considered activities with individuals talking about their profession and the items were limited to questions about how the BHF Resources would fit within their work or community activities, rather than about the individual characteristics of the interviewee, this work was not considered human subjects research by the University of Nebraska Medical Center IRB. However, while informed consent was not required; the research team asked all interviewees for permission to record the interview so that an accurate analysis of interview responses mapped to business model hypotheses could be completed. Interviews were conducted using phone or video conferencing and recorded using a password-protected, data-driven product management software platform that allowed for research team members to review interviews and feedback, track new discoveries, and validate (or refute) business model hypotheses. Upon completion of each interview, participants were asked for contact information of others in their network that could provide insight on their experiences addressing childhood obesity and the organizational decision-making process around adopting new evidence-based interventions.

### Data analysis

A unique aspect of our approach to customer discovery interviews was the use of a rapid qualitative reduction process to analyze the research team members’ notes from each interview [26]. During this process, the research team member entered major themes from the interview into an online database and reviewed the existing hypotheses to code the interview as confirming, leaning confirming, neutral, leaning disconfirming, disconfirming, or not applicable to each hypothesis. Codes were discussed by research team members to ensure consensus. For example, if an interview yielded a major theme of fostering better clinic-community partnerships to allow for more referrals to effective FHWPs, the research team member would code that interview as “confirming” for the hypothesis that community communication and marketing resources would be a direct benefit to the customer (value proposition). Similarly, the same interview may have revealed that community health improvement plans are a contributing factor, but not the sole driver of program adoption decisions, and therefore the interview would be coded as “neutral” for the hypothesis, community health improvement plan goals are a primary driver of resource adoption (value proposition). The protected time provided by the research team’s involvement in the SPRINT program allowed for this more thorough data analysis process than what may be employed in more traditional customer discovery activities [19]. Codes for each hypothesis were quantified and used to rank order the value propositions.

Throughout the customer discovery interviews and expert consultation with the SPRINT facilitation team, we made changes to our hypotheses across all elements of our business model. If participant responses did not align with existing hypotheses, new hypotheses were created in the business model and added to subsequent interview guides. The research team met weekly to review interview progress, refine our target audience, and to discuss business model hypotheses. Hypotheses were edited, created, or removed (an activity prescribed by the customer discovery process) to reflect findings from the interviews and better capture the organizational decision-making process around adopting new evidence-based interventions. Across interviews and team discussions, emergent themes that could inform future scale up were recorded.

## Results

### Customer discovery

Participants in the customer discovery interviews and the expert SPRINT facilitators indicated that identifying a specific type of community organization that was likely available across a wide variety of micropolitan and rural communities may be necessary to improve marketing and dissemination strategies for BHF program facilitators – the primary end users of the BHF Resources Package. Through the interviews, the consistent type of community organization mentioned was local health departments, and therefore potential BHF program facilitators were indicated to be local health department directors who have decision-making authority over local programming within the broader context of community health priorities. In contrast to identifying a potential single community organization for BHF implementation, interview participants identified a wide range of secondary end users (i.e., those who would support or provide funding for communities to use the BHF Resource Package). The potential supporters or funders included local employers, hospitals, and nonprofit organizations that could provide financial support for local health departments interested in adopting, implementing, and sustaining BHF.

### Value propositions

Over the course of the customer discovery process, we identified nine components of value propositions related to why a customer would adopt the BHF product, how the resource would be a direct benefit to the customer, or what is unique about our solution. These are rank ordered in Table 2 based upon the degree to which they were confirmed across interviews. Specifically, based on confirming and leaning confirming responses, the most consistently confirmed value proposition across interviews was the hypothesis that communities will value the communication materials and marketing resources included in the BHF Resource Package. By rank order of confirmation, the next three value propositions all addressed the alignment of the BHF Resource Package with community priorities and need. Interviewees valued the focus on interorganizational collaboration promoted by the BHF Resource Package. While confirmed, our initial value hypothesis – that communities would value the user-friendly, comprehensive training and resource package – was rank ordered sixth in the nine value proposition hypotheses. Finally, our value hypothesis that the BHF Resource Package would be valued because it would support facilitator work by aligning with current job responsibilities was not confirmed.

**Table 2:** Value proposition hypotheses related to the BHF Resources Package in order of strength of qualitative confirmation through customer discovery interviews

1. Communities will value the communication materials and marketing resources included in the BHF Resource Package.
2. The BHF Resource Package will help meet community health improvement plan goals.
3. The BHF Resource Package will fill the need for non-clinical treatment options in communities.
4. There will be synergy between organizational missions and BHF Resource Package goals.
5. Communities will value the encouragement for interorganizational collaboration with the BHF Resource Package.
6. Communities will value the user-friendly, comprehensive training/resource package.
7. Communities will value the cultivation of clinical support to deliver BHF.
8. Social benefits and cross-community relationship development will be valued by potential payers.
9. Alignment of BHF with current job responsibilities will be valued by implementation organizations.

### Emergent themes from interviews

Findings from our customer discovery interviews illuminated insights beyond identifying local health departments as our primary customer. These themes were present across our business model hypotheses and included: 1) perceptions of responsibility among clinical and community organizations; 2) payer opportunities; and 3) marketing the benefits of the BHF Resource Package beyond child weight change.

#### Perceptions of responsibility among clinical and community organizations

We interviewed medical providers and clinical staff (*n*=10) as well as health practitioners within the community (i.e., health department staff; *n*=13). What emerged from these discussions was that lifestyle programming for childhood obesity falls into a “grey area” between clinic and community systems. This also provided the context for the finding that there was a lack of alignment between the responsibilities of implementing BHF and current job descriptions of those working in micropolitan and rural communities. Specifically, several of the interview participants discussed the gap in rural communities – between which FHWPs fall – where local health departments focus on obesity prevention and rural health centers do not have the capacity to deliver FHWPs. Thus, FWHP implementation was not a consistent or specific job responsibility in rural communities. Within the potential BHF Resource Package user group, health department interviewees indicated that childhood obesity represented a clinical concern and that approaching families about childhood obesity treatment falls outside of the perceived scope of community health professionals. In contrast, healthcare professionals who were interviewed highlighted the constraints in clinical time and resources needed to deliver recommended FHWPs. Of note, interviewees suggested that there may be the potential for the public health and healthcare systems to complement one another to address childhood obesity by leveraging the patient-provider relationship to refer patients to efficacious community-based programming.

#### Cost, revenue, and payer opportunities

A common theme during the customer discovery interviews was the cost of providing the program and possible funding and reimbursement structures to support the sustainability of a FHWP. Interviewees identified potential grant funding or sponsorship from local or national foundations, local healthcare community benefit dollars, and employers as the most likely sources of sustainable funding for BHF. Additionally, licensing fees for adopting organizations or advertisement opportunities to offset costs, and a tiered system of customer fees (i.e., participating families) were proposed by interviewees as potential funding mechanisms. A consistent scenario was described that included a combination of a licensing fee for community organization use of the BHF Resources Package, additional fees for technical support or other methods to facilitate community organization adoption and implementation, and a small fee for participating families based on ability to pay. Finally, interviewees also discussed the potential of insurance reimbursement for family participation in BHF, similar to the reimbursement structure in place for the CDC National Diabetes Prevention Program.

#### Marketing the benefits of BHF beyond child weight change

As the BHF Resources Package is a FHWP, we designed the marketing and recruitment materials to highlight the health promotion aspects of BHF, specifically the focus on behavior change strategies that promote a healthy lifestyle and weight management. However, during our interviews, participants consistently emphasized the health promotion benefits of the BHF program that extend beyond weight management. Additional benefits included, but moved beyond, changes in healthful eating and physical activity to the constructive use of family time, social and emotional well-being, and quality of life. Participants explained that adopting a program like the BHF Resources Package would be more feasible if it could easily be identified as meeting several organizational health goals within nutrition, physical activity, and social and emotional well-being domains. Responses from health department professionals specifically referred to BHF aligning with Community Health Improvement Plans as being an important consideration. Finally, some interviewees highlighted the benefits of providing BHF in workplaces to promote employee well-being and reduce turnover.

## Discussion

Using a customer discovery process provided several critical areas for consideration when addressing the potential adoption, implementation, and sustainability of FHWPs in micropolitan and rural communities. Specifically, our findings suggested that there is a need to not only focus, but expand on the characteristics of an innovation related to designing for dissemination and scalability. Further, we identified the value held by potential delivery organizations in the resources that address issues related to reaching those in need of FHWPs and support for interorganizational collaboration. Finally, we found there is a need to address the ongoing challenges of the grey area of implementation responsibility for FHWPs and how that influences the availability of potentially sustainable funding models.

The BHF Resources Package, was designed for dissemination using a participatory and user-centered approach to create a resource that included all the program materials, training guides, and evaluation materials that micropolitan, rural, and/or under resourced communities need to implement BHF [10]. As such, we anticipated that the customer discover process would underscore that potential users of the BHF Resource Package would highly value the user-friendly comprehensive training and resource package features. This is consistent with much of the reporting on previous research teams that have used the customer discovery process that emphasizes the fit between the characteristics of the evidence-based intervention and delivery setting [3]. Our customer discovery process identified this as a value to micropolitan and rural communities, however, the relative rank of our value proposition related to designing for dissemination was rated sixth out of our nine proposed value statements. This may indicate that the user-friendly, comprehensive BHF Resource Package may be a necessary, but insufficient characteristic that communities consider in the adoption decision making process. Specifically, factors that are related to, but distinct from the characteristics of BHF Resource Package are the degree to which the goal of FHWPs aligns with community priorities, organizational missions, and degree of inter-organizational collaboration.

There are two areas in our findings that have not been explicitly addressed in other reports from the customer discovery process within the SPRINT process. First, the highest value that interviewees found in the BHF Resource Package was the inclusion of communication materials and marketing resources intended to increase the reach of the FHWP. Second, we found that interviewees also valued support for interorganizational collaboration. These findings may be due to the unique focus on micropolitan and rural communities, and align with previous research examining FHWPs in micropolitan and rural areas, where members of a community advisory board highlighted the development of sustainable referral protocols that relied on engagement across community organizations [1].

Perhaps the most daunting challenge identified through the customer discovery process is the grey area of responsibility when it comes to implementing FHWPs and the seemingly conflicting feedback we received that local health departments should be the focus of adoption efforts, but that health departments also prioritize obesity prevention over obesity treatment. Unfortunately, this is not new to our research team and is the underlying rationale for why we approached the development of the BHF Resources Package with a goal to allow for delivery by different organizations based on local systems that prioritized family healthy weights [11]. Still, focusing on local health departments served as a compass for steering our discussions with prospective partners who were also included in our customer discovery interviews and led to an approach that still valued flexibility in a local implementer while including local health departments as the primary starting place. In addition, after our completion of the customer discovery process, the CDC High Obesity Program included the implementation of FHWPs through state health departments [2] which should improve the market pull for the BHF Resource Package.

Moving forward with the intention to scale up the BHF Resource Package has been substantially informed by the findings from our customer discovery interviews and SPRINT training. Specifically, this process led to the development of more tailored descriptions of the BHF Resource Package that align with the values highlighted by our interviewees. A primary adaptation to the BHF Resource Package as a result of both the customer discover interviews and our piloting of the BHF Resource Package, was to enhance the recruitment module, introduce training for a recruitment coordinator, and provide a protocol to develop a local recruitment team. We anticipate this adaptation will be well received by communities. Yet, like other health promotion strategies intended for community, identifying potential payers for community-based lifestyle programming and moving towards commercialization is an ongoing and evolving challenge [15, 19]. At the completion of this project, the goal of the research team was to develop a non-profit business, embedded within a host university, to provide the ability to receive payment from communities interested in using the BHF Resource Package. Challenges with this process included the time necessary to complete the process within the context of maintaining team project management, research, teaching, and service responsibilities [19]. Further, as we have moved forward, we have found that understanding the actual costs to provide the BHF Resource Package –from initiating a contract with a delivery organization to providing ongoing technical support—to be a challenge. As such, we recommend that others who are examining the potential to scale interventions like the BHF Resource Package consider using simple contractual agreements with a small number of implementation sites to better understand the costs prior to initiating commercialization processes—nonprofit or otherwise.

This project is limited by the focus specifically on micropolitan and rural communities primarily in Nebraska and the surrounding states. The findings may not be generalizable to other populations or systems outside of this region. For example, in our region, systems such as cooperative extension do not focus on childhood obesity treatment, though in other regions they do [13, 14, 21, 22] and may be another potential primary user of the BHF Resource Package (or other FHWPs). This underscores the potential need to consider the variability of organizations and resources to deliver evidence-based FHWPs if a broad public health impact in rural areas is to be achieved. Finally, when contrasted with other projects that have used customer discovery processes with the SPRINT model, it appears that there are unique issues related to promoting family healthy weights in micropolitan and rural communities.

## Data Availability

The datasets used and/or analysed during the current study are available from the corresponding author on reasonable request.

## Abbreviations

AAP: American Academy of Pediatrics
BHF: Building Healthy Families
CDC: Centers of Disease Control and Prevention
FHWP: Family Healthy Weight Program
RE-AIM: Reach, Effectiveness, Adoption, Implementation, and Maintenance Framework
SPRINT: SPeeding Research-tested INTerventions
